# Multi-carbon dots and aptamer based signal amplification ratiometric fluorescence probe for protein tyrosine kinase 7 detection

**DOI:** 10.1186/s12951-021-00787-7

**Published:** 2021-02-15

**Authors:** Yunsu Ma, Yuan Wang, Yongjie Liu, Lujia Shi, Dongzhi Yang

**Affiliations:** School of Pharmacy, Xuzhou Medical University, Xuzhou, 22004 Jiangsu People’s Republic of China

**Keywords:** Ratiometric fluorescence, PTK 7, Carbon dots, Signal amplification, Aptamer

## Abstract

**Background:**

Protein tyrosine kinase 7 (PTK 7) is a membrane receptor, which can be found in various kinds of cancers. In view of this, detection of PTK 7 in the peripheral circulation would be an effective way for the early diagnosis of cancer.

**Results:**

In this work, a multi-carbon dots and aptamer-based signal amplification ratiometric fluorescence probe was developed. The fluorescence of the aptamer-modified y-CDs and b-CDs were respectively chosen as the detection signal and interior label. The fluorescence of y-CDs was quenched by Fe_3_O_4_ and cDNA (complement to aptamer) compound without PTK 7, but recovered by the addition of PTK 7. Then, the free aptamer was cut by DNase I, which amplified the detection signal. The ratiometric fluorescence sensor for PTK 7 was established with the LOD of 0.016 ng mL^−1^.

**Conclusions:**

Summary, a multi-carbon dots and aptamer-based signal amplification ratiometric fluorescence probe was developed for the detection of protein tyrosine kinase 7. The developed probe was applied to PTK 7 detection in MCF-7 cells and human serum with satisfying results, thus indicating that this probe has huge potential in clinical practice.

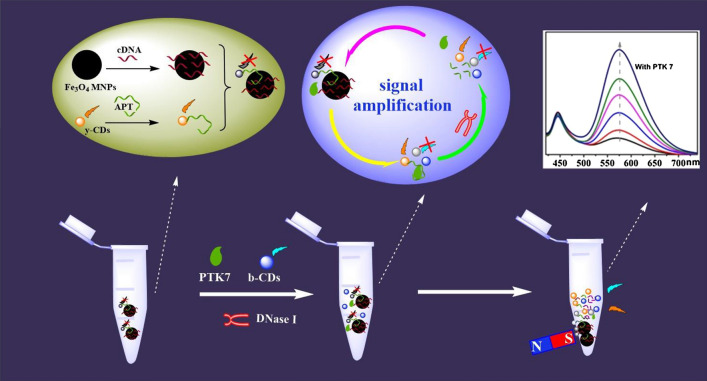

## Background

Cancer, as the disease with the highest mortality rate, attracts a huge amount of attention and during these pressing moments, the early diagnosis of cancer plays an important role in its therapy [1–3]. The detection of biomarkers in body fluid (like blood, urine, saliva) provide a safe and effective way for the early diagnosis of cancer [4–6]. Protein tyrosine kinase 7 (PTK 7), also known as colon cancer kinase-4, is a membrane receptor which is encoded by the PTK 7 gene, which modulates multiple Wnt pathways [7]. As stated in previous reports, PTK 7 is overexpressed in various tumours, such as in lung cancer, gastric cancer, ovarian cancer and colon cancer and thus, the detection of PTK 7 in the peripheral circulation is a new way to make an early diagnosis [8]. It is essential to find a sensitive, selective, safe and simple way to detect PTK 7. Several analytical methods have been reported in previous research, including western blotting, flow cytometry, enzyme-linked immunosorbent assay, the fluorescence analysis method and electrochemistry immunoassay [9], among which the fluorescence analysis method has been widely used in various fields by virtue of its fast response time, low cost, simple usage and high sensitivity [10]. Therefore, the fluorescence method is perceived to be one of the most promising analytical tools for PTK 7 detection.

Nanomaterials have been wildly used in biomedical application [11–15]. Carbon dots (CDs) are a new kind of fluorescence nanomaterial that have attracted wide attention owing to their excellent properties including good photostability, easy modification, stable chemical properties, favourable biocompatibility and excellent water-solubility [16, 17]. Some of their properties, specifically for metal ions, micromolecules and protein analysis, have meant that CDs have been used to set up biosensors [18–20]. Because of the relatively low concentration of PTK 7 in peripheral circulation, the biosensors for PTK 7 detection need to be equipped with high sensitivity and selectivity. However, despite possessing excellent optical performances, the fluorescence materials were found to be limited during the development of the PTK 7 sensor as they lacked good selectivity. Working from this base, an aptamer was introduced to develop a fluorescence probe. In addition to this, the loop amplification strategy was employed to enhance the sensitivity of that developed probe. Now, with these additions in place, the selectivity of PTK 7 detection was effectively increased.

Aptamers are DNA or RNA oligonucleotides, which are selected from random-sequence nucleic acid libraries by an exponential enrichment (SELEX) process, and can specifically bind themselves to target molecules through base pairing [21]. The advantages that aptamers have of being low-cost, and having high stability, and easy synthesis and labelling mean that aptamers are favoured when building biosensors [22–24]. Following this introduction of an aptamer, a significant improvement was seen in the selectivity of the fluorescence probe. As previously reported, an aptamer-based fluorescent platform for ultrasensitive adenosine was developed with a 60 PM limit of detection (LOD) [25]. Furthermore, given that the aptamer is a single-stranded RNA or DNA that can be cut by the restriction enzyme, it became possible to introduce rolling circle amplification into the development of the biosensor. As Yao and his co-workers reported, a photonic crystal-assisted biochip was constructed using this rolling circle amplification to circulate microRNAs detection in serum with an LOD of 0.7 aM [26]. The strategy of nucleic acid amplification was also successfully used in the construction of the fluorescence probe for microRNAs [27]. Additionally, in fluorescence sensors, the ratiometric fluorescence measurement can afford simultaneous recordings of two measurable signals at one excitation wavelength [18, 28]. This is because one measurable signal plays the role of the interior label, which can then overcome the drawback that is the ease in which a single fluorescence measurement can be influenced by the detection conditions and probe concentrations [29, 30]. Nonetheless, CDs are rarely used in ratiometric fluorescence biosensor for PTK 7 detection. Taking into account the excellent properties of CDs, it is essential to build a ratiometric fluorescence probe for the detection of PTK 7.

Herein, we aimed to develop a sensitive, selective and simple fluorescence sensor for the detection of PTK 7. Dual carbon dots, which emit blue and yellow fluorescence (b-CDs and y-CDs) and Fe_3_O_4_ MNPs, were chosen to build a ratiometric fluorescence probe. As designed, the fluorescence of y-CDs (modified by the aptamer of PTK 7) worked as the detection signal which was then quenched by the Fe_3_O_4_ magnetic nanoparticles (MNPs) and cDNA (complement to aptamer) compound. The fluorescence of b-CDs was the interior label, and Fe_3_O_4_ MNPs were used for magnetic separation. When encountering PTK 7, the fluorescence produced by the y-CDs was recovered by separating it from Fe_3_O_4_ MNPs. In addition to this, the detection signal was loop amplified by adding DNase I. So, a ratiometric fluorescence sensor towards PTK 7 was established by measuring the fluorescence signals from the y-CDs and b-CDs. The developed probe was applied to the determination of PTK 7 in MCF-7 cells and human serum to ensure its effective application.

## Experimental section

### Chemical and reagents

Ferric trichloride, sodium acetate, citric acid monohydrate, diethylenetriamine, *N*-(3-dimethylaminopropyl)-Nethylcarbodiimide hydrochloride (EDC), *N*-hydroxysuccinimide (NHS), 4-aminobutyric acid (GABA) and *o*-phenylenediamine (OPD) were purchased from Sinopharm Chemical Reagent Co., Ltd. (Shanghai, China). Recombinant Protein Tyrosine Kinase 7 (PTK 7) was obtained from Wuhan USCN Business Co., Ltd. (Wuhan, China). The base sequences of the DNA oligonucleotides were provided as follows: aptamer of PTK7 (APT, ATC TAA CTG CTG CGC CGC CGG GAA AAT ACT GTA CGG TTA GA), cDNA (complementary to part of the PTK7 aptamer, TCT AAC CGT ACA GTA TTT TCC CGG CGG CG), and all of them were purchased from Shanghai Sangon Biotechnology Co., Ltd. (Shanghai, China). *E. coli* exonuclease I (DNase I, > 10 U μL^−1^) was also provided by Shanghai Sangon Biotechnology. The purified water was prepared using a water purification system that was obtained from the Thermo Scientific technology Co., Ltd. (Shanghai, China). All other chemicals that did not have special specifications but were used in this paper were of analytical grade and used without further purification.

### Measurement and apparatus

The fluorescence emission spectra shown in this paper were measured on a F4600 spectrofluorometer (Hitachi, Japan) with 380 nm excitation. FT-IR spectra and UV–vis spectra were respectively recorded on a FTIR-8400S spectrometer (Shimadzu, Japan) and WFN-203B spectrometer (Jingke, China). The transmission electron microscopic images (TEM) were measured by a JEM-2100 transmission electron microscope (JEOL, Japan). The size distribution of nanoparticles was determined by the Nicomp380ZLS dynamic light scattering technique (Santa Barbara, USA).

### Synthesis of Fe_3_O_4_ magnetic nanoparticles (Fe_3_O_4_ MNPs)

Fe_3_O_4_ MNPs were synthesised according to previously reports [31]. Briefly, 0.54 g of FeCl_3_ and 1.44 g of sodium acetate were dissolved in 10 mL of glycol. The mixture was formed by adding the sodium acetate solution to the FeCl_3_ solution dropwise, while mixing it with a magnetic stirrer. Then, the mixture was moved to a Teflon-lined stainless-steel autoclave and heated at 200 °C for 8 h. The Fe_3_O_4_ magnetic nanoparticles were obtained after the reacted mixture was washed with ethanol 3 times. Subsequently, 0.2 g of dry Fe_3_O_4_ magnetic nanoparticles were added to 50 mL of citric acid monohydrate solution (0.1 g mL^−1^) and placed under an ultrasound for 20 min. Then, the mixture was mixed with a magnetic stirrer for 4 h at room temperature. The products of the reaction were washed with water and ethanol several times, dried under vacuum at 50 °C, and then the carboxylate Fe_3_O_4_ MNPs were prepared.

### Synthesis of Fe_3_O_4_-cDNA

First, cDNA was centrifuged at 4000 rpm for 1 min, and then 25 uL of phosphate buffer (PBS, pH = 7.5) was added. Subsequently, cDNA (100 μM) was heated at 95 °C for 4 min, cooled by ice bath for 4 min, and after that, left to rest at room temperature for a while [32]. This could facilitate folding cDNA into the minimum energy structure, which is responsible for specific binding. Second, a mixture of EDC (10 mg) and NHS (10 mg) was added into 1 mL of Fe_3_O_4_-COOH solution (0.2 mg mL^−1^), which was then dissolved using PBS (pH = 6.0). The mixture was stirred at 25 °C for 30 min, and the pH was adjusted to 7.5. Finally, 10 μL of the pre-processed cDNA (100 μM) and 1 mL of Fe_3_O_4_–COOH was mixed and continuously stirred at room temperature overnight to conduct the amide linkage between the carboxyl and amino groups. Fe_3_O_4_-cDNA was obtained through the use of magnetic separation and washed several times to remove any unreacted cDNA. The prepared Fe_3_O_4_-cDNA was stored in PBS (pH = 7.5) at 4 °C for further use.

### Synthesis of b-CDs and y-CDs

The b-CDs were prepared using the one-pot hydrothermal method, as previously reported in the literature [33]. Briefly, using an ultrasonic method for a period of 10 min, 1.2 g of citric acid and 600 μL of diethylenetriamine were dissolved in 20 mL of ultrapure water. Then, the mixture was poured into a Teflon-lined stainless-steel autoclave and heated at 200 °C for 6 h. After having cooled to room temperature, the product was collected and dialysed. Afterwards, using the vacuum drying method, the brown powder was obtained for further characterisation and research. The y-CDs were prepared by the same method as b-CDs [34]. We used 0.3 g o-PD and 0.3 g GABA as the carbon source, which were then dissolved in 20 mL of ultrapure water and sonicated for 15 min. The mixture was moved to a Teflon-lined stainless-steel autoclave, heated at 160 °C for 6 h, and then cooled to room temperature. The brownish yellow solution was obtained after it had been dialysed and enriched using the same method used for the preparation of b-CDs. The b-CDs and y-CDs were stored at room temperature for further research.

### Synthesis of y-CDs-APT

To prepare y-CDs-APT, the carboxyl groups of y-CDs were activated by EDC and NHS. 1 mL y-CDs (0.1 mg mL^−1^, pH = 6.0), 10 mg EDC and 10 mg NHS were mixed and stirred at room temperature for 30 min, and then the pH of the mixture was adjusted to 7.5. In this part, APT was processed using the same method as cDNA, before then being added to y-CDs. Then, 10 μL APT was incubated with 1 mL y-CDs (100 μM) at room temperature for the night to allow for the synthesis of y-CDs-APT. After that, the prepared y-CDs- APT was stored at 4 °C for further research.

### PTK 7 detection

For the detection of PTK 7, 250 μL of the prepared Fe_3_O_4_- cDNA and 250 μL of the prepared y-CDs-APT were mixed and then incubated at 25 °C for 60 min. The mixture was then washed 3 times using PBS by magnetic separation. Following that, 600 μL variable concentrations of PTK 7, 20 μL b-CDs (1.00 ng mL^−1^) and 10 μL DNase I (20 U μL^−1^) were added into the resulting solution, and incubated at 37 °C for 30 min. Then, the supernatant of the resulting solution was collected through magnetic separation, and the fluorescence spectra were measured under 380 nm excitation.

### Real sample determination

To discover the feasibility and practicality of this developed ratiometric fluorescence probe, two types of real samples (MCF-7 cells and human serum) were determined. Firstly, MCF-7 cells were incubated at 37 °C and with 5% of CO_2_ for 24 h, and then they were seeded into a plate at various densities (1 × 10^4^, 2.5 × 10^4^, 5 × 10^4^, 1 × 10^5^, 2.5 × 10^5^ and 5 × 10^5^ per mL). After 4 h of incubation, 600 μL of MCF-7 cells at various densities were detected, as described in “2.7 PTK 7 detection”, followed by the implementation of the standard addition method to verify the reliability of the MCF-7 cells determination. For the detection of PTK 7 in human serum samples however, the human serum was diluted with PBS by a factor of 400. Then, the operation was carried out according to section “PTK 7 detection.” The reliability of the serum sample detection was also verified using the standard addition method.

## Results and discussion

### Materials characterisation

**Fig. 1:**
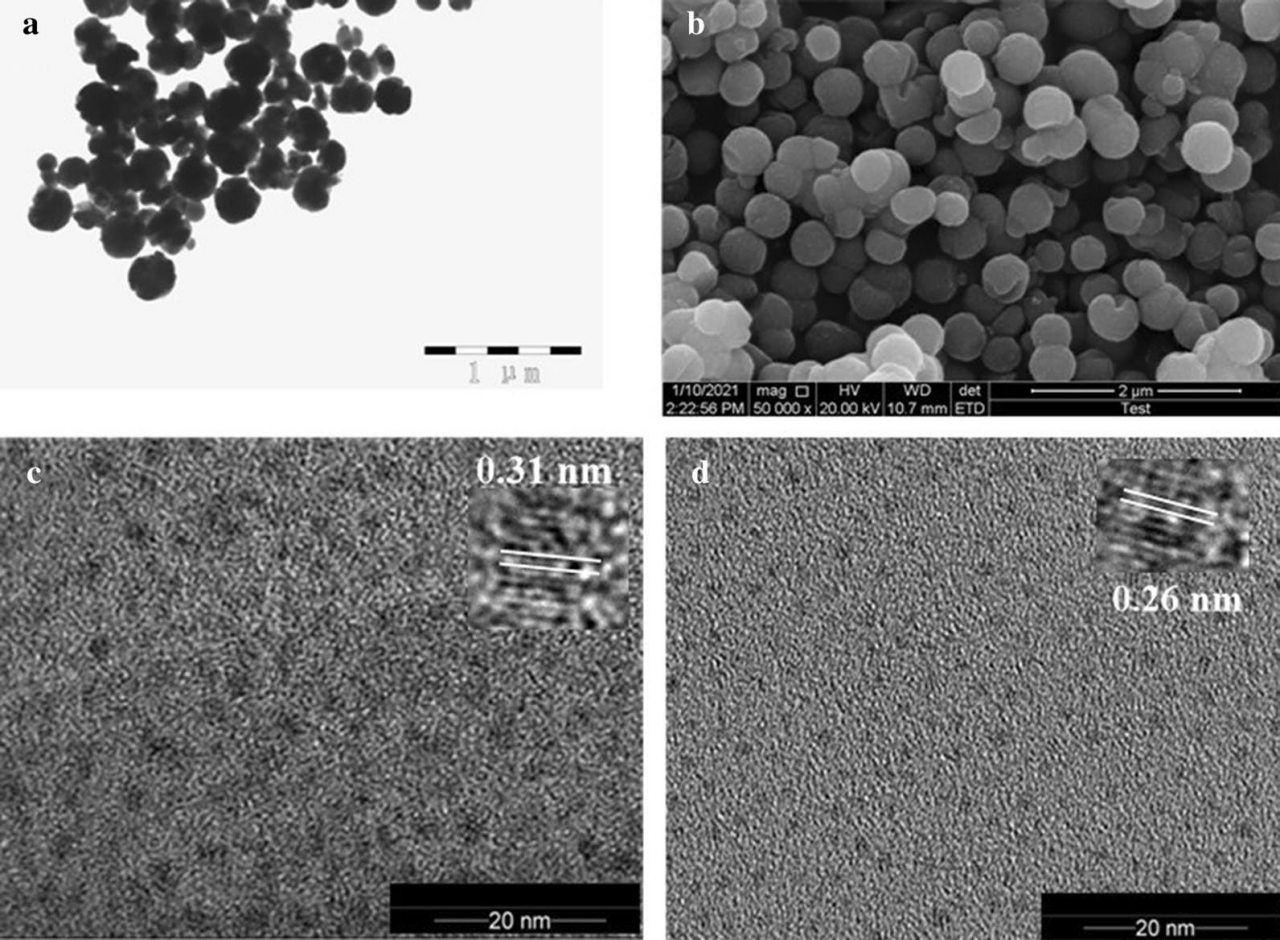
**a** The TEM images of Fe_3_O_4_ MNPs. **b** The SEM images of Fe_3_O_4_ MNPs. **c** The TEM image of b-CDs, Insert: high-resolution TEM image of b-CDs. **d** The TEM image of y-CDs, Insert: high-resolution TEM image of y-CDs

The TEM images of nanoparticles are displayed in Fig. 1. As shown in Fig. 1a, the Fe_3_O_4_ MNPs presented a rounded shape with a diameter of about 200 nm, and they dispersed well in the water solution [29]. The SEM images of Fe_3_O_4_ MNPs in Fig. 1b also displayed that they were evenly dispersed with spherical shape, which consistent with the TEM results. The TEM images of b-CDs and y-CDs were shown in Fig. 1c and d, respectively [16, 35]. As shown, b-CDs were bigger, with a lattice fringe of 0.31 nm (insert of Fig. 1c), while y-CDs had a lattice fringe of 0.26 nm (insert of Fig. 1d). Both of them showed a spherical shape and were evenly dispersed when in aqueous solution.

**Fig. 2:**
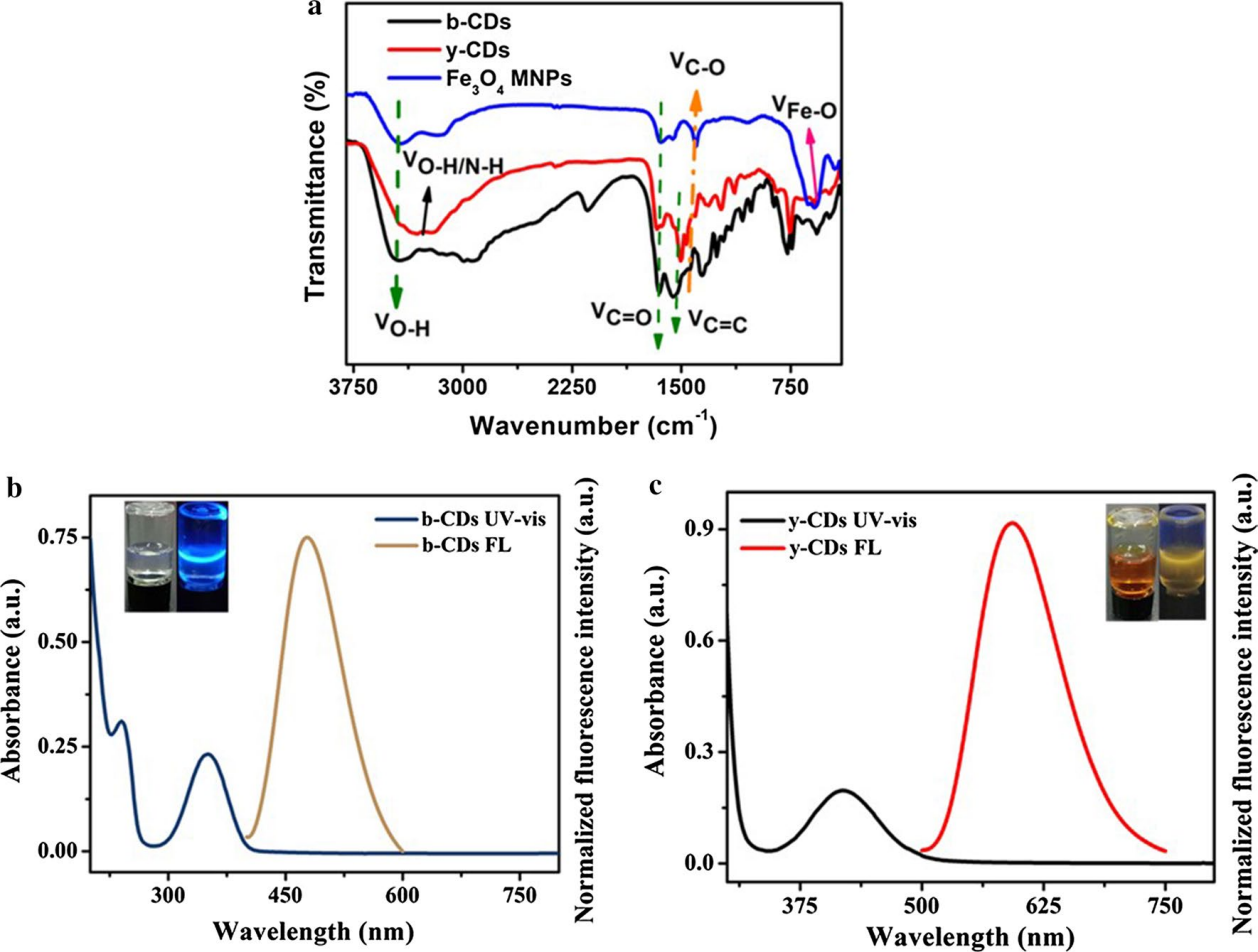
**a** The FT-IR spectra of b-CDs, y-CDs and Fe_3_O_4_ MNPs. **b** The UV–vis absorption spectrum and fluorescence spectrum of b-CDs. Insert: the solution of b-CDs under day light and UV-lamp, respectively. **c** The UV–vis absorption spectrum and fluorescence spectrum of y-CDs. Insert: the solution of y-CDs under day light and UV-lamp, respectively

The FT-IR spectra were measured for the purpose of characterising the structures of the nanomaterials used in this study. As shown in Fig. 2a, the broad vibration band around 3400 cm^−1^ was ascribed to –OH stretching vibration, while the peaks at 1680 and 1200 cm^−1^ were ascribed to C=O and C–O stretching vibration, which appeared in the FT-IR spectra of b-CDs, y-CDs and Fe_3_O_4_ MNPs [36, 37]. It can be summarised from the above stated results that there were carboxyl groups on their surface. For b-CDs and y-CDs, the FT-IR spectra also showed peaks at 1580 and 1490 cm^−1^, which were attributed to the C=C vibrations in the benzene ring, indicating that there were aromatic structures in both b-CDs and y-CDs [22, 25]. The broad vibration band around 3300 cm^−1^ and the peak at 1450 cm^−1^ were attributed to the vibrations of O–H/N–H and C-N, which thus implied an existence of –NH_2_ in y-CDs [34]. For Fe_3_O_4_ MNPs, the stretching vibrations of Fe–O showed a characteristic peak near 740 cm^−1^ in the FT-IR spectra [31].

The UV–vis absorption spectra and fluorescence spectra were measured to further study the characterisation of b-CDs and y-CDs. The UV–vis absorption spectrum of b-CDs (Fig. 2b) exhibited two obvious absorption bands at ~ 260 nm and ~ 340 nm, which resulted from π–π* transition of sp^2^ carbon and n–π* transition of C=O/C=N in b-CDs, which were in accordance with the FT-IR results [35, 38]. As shown in Fig. 2b, the fluorescence spectrum of b-CDs displayed a sharp peak at 445 nm, which was due to being excited at the wavelength of 380 nm. The insert images in Fig. 2b show the respective solution of b-CDs under day light and UV-lamp. As shown, it was colourless when under day light but had blue emissions when under the UV-lamp. The UV–vis absorption spectrum of y-CDs also exhibited two obvious absorption bands at ~ 260 nm and ~ 410 nm in Fig. 2c. The peak at ~ 260 nm was considered as π–π* transition caused by sp2 domains in the core of y-CDs, while the wide absorption band at ~ 410 nm could be denoted as the functional groups, like C=O/C=N, which were attached to the surface of y-CDs [16, 34]. The fluorescence of y-CDs had a yellow emission, and when excited at 380 nm the fluorescence spectrum showed an emission wavelength at 575 nm. In addition, the insert images in Fig. 2b showed that the solution of y-CDs was brown when under day light but had a yellow emission when under UV-lamp.

### Principle of PTK 7 sensing

**Scheme 1:**
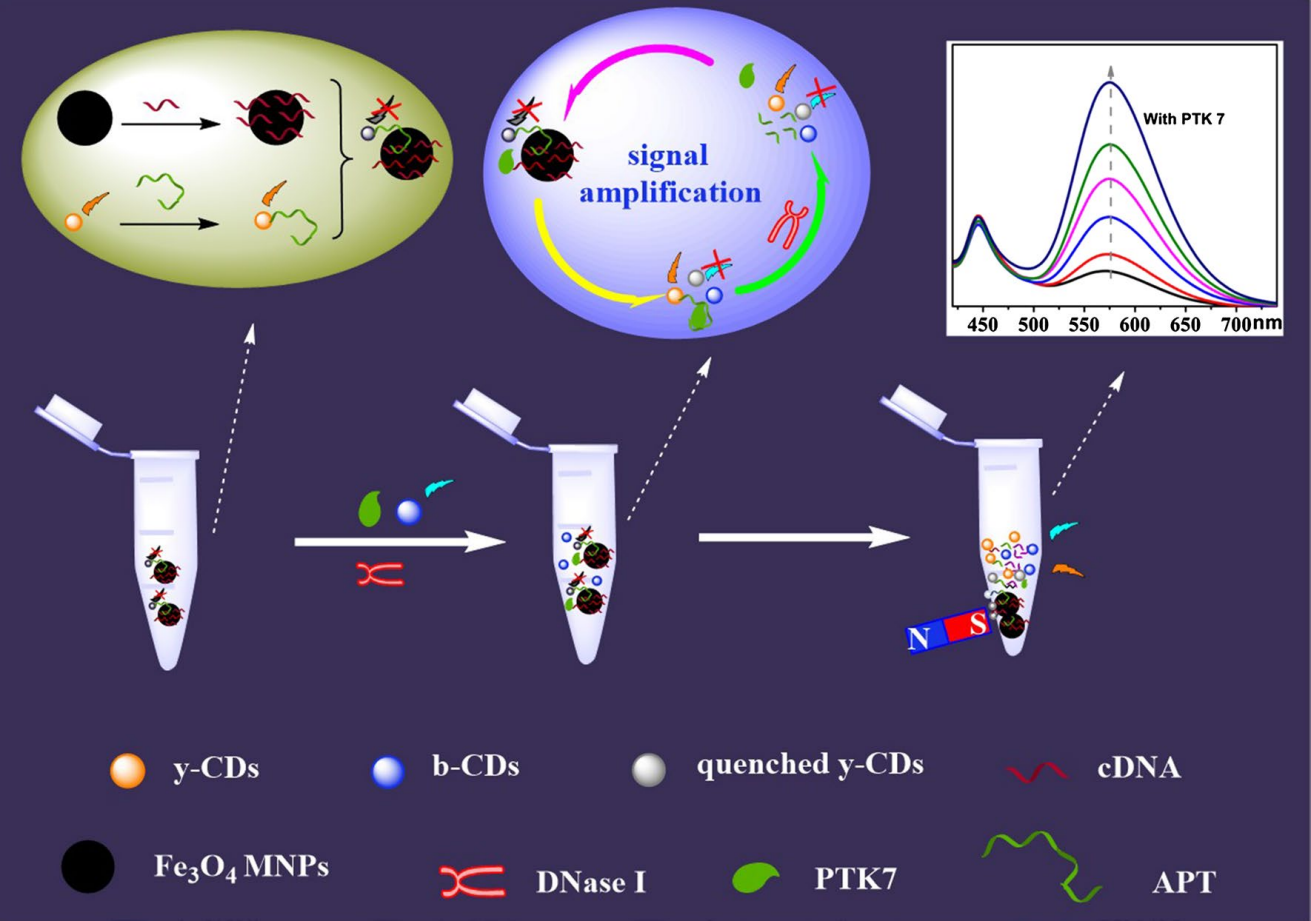
Preparation process and PTK 7 detection mechanism of the ratiometric fluorescence probe

In this paper, a ratiometric fluorescence probe for the detection of PTK 7 was developed based on dual carbon dots and then it was applied to real samples to test its effectiveness. The preparation process and PTK 7 detection mechanism of this ratiometric fluorescence probe is described in Scheme 1. Firstly, cDNA (complementary to part of the PTK7 aptamer) was connected with Fe_3_O_4_ to form Fe_3_O_4_-cDNA, and APT (aptamer of PTK7) was conjugated with y-CDs to form y-CDs-APT. The fluorescence of y-CDs was quenched by the specific connection between Fe_3_O_4_-cDNA and y-CDs-APT (y-CDs-APT-cDNA-Fe_3_O_4_). The fluorescence of y-CDs was then recovered after adding PTK7, as y-CDs-APT conjugated with PTK7 (y-CDs-APT-PTK7) to isolate it from Fe_3_O_4_-cDNA. Then, the APT of y-CDs-APT-PTK7 was cut by adding DNase Ι, which resulted in the freeing of PTK7. Subsequently, the y-CDs-APT-cDNA-Fe_3_O_4_ was broken once more by the free PTK7, more y-CDs were removed from the surface of Fe_3_O_4_, and a loop amplifier was developed. In summary, the detection signal of this ratiometric fluorescence probe was provided by y-CDs and b-CDs, and was amplified by DNase Ι.

**Fig. 3:**
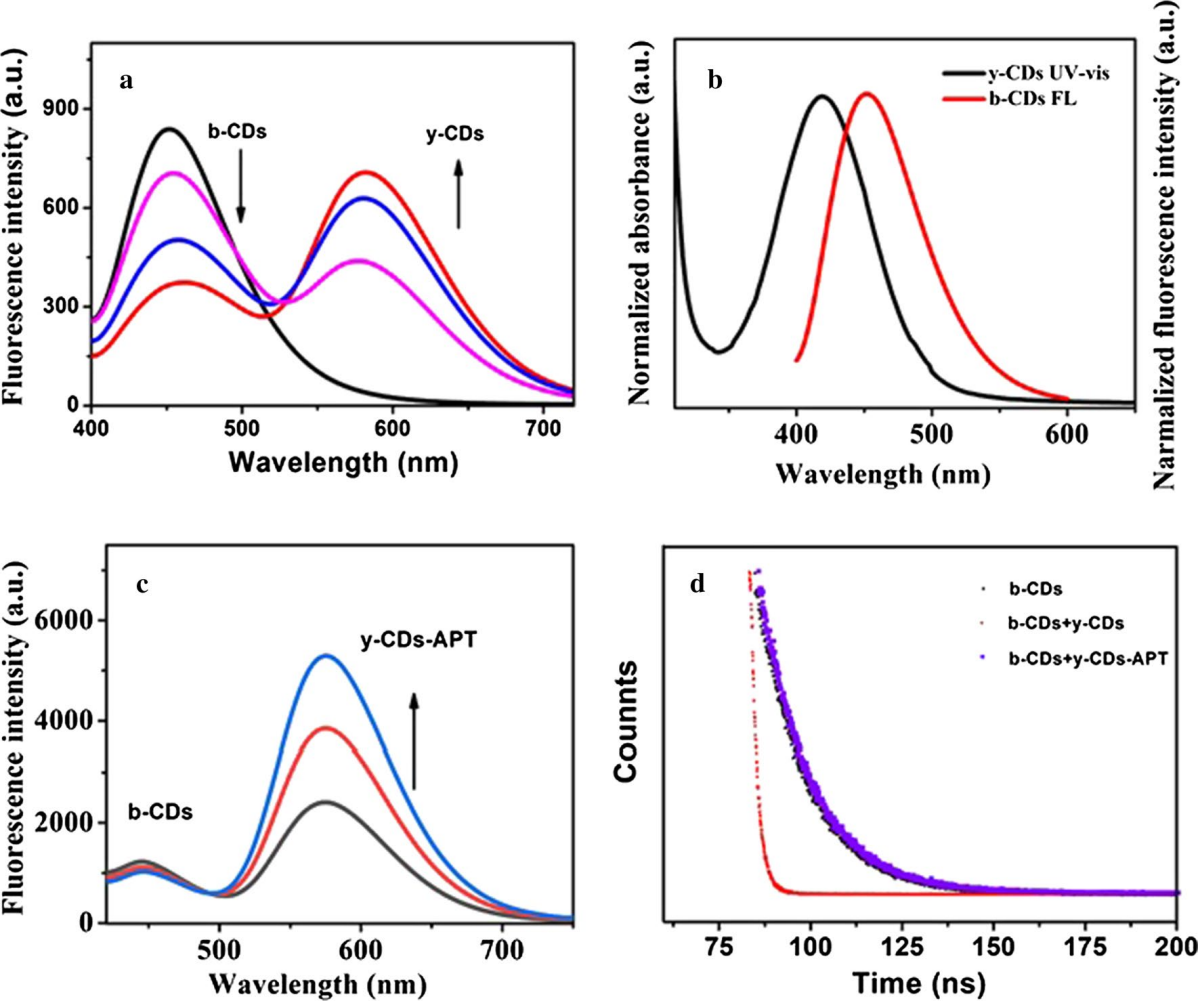
**a** The fluorescence spectra of b-CDs mixed with various concentrations of y-CDs. **b** The UV–vis absorption spectrum of y-CDs and the fluorescence spectrum of b-CDs. **c** The fluorescence spectra of b-CDs mixed with various concentrations of y-CDs-APT. **d** The fluorescence lifetime spectra of b-CDs, b-CDs + y-CDs and b-CDs + y-CDs-APT

To discover a possible detection mechanism for this probe, various research papers have been studied. Obviously, the inner-filter effect based on the extensive ultraviolet absorption of Fe_3_O_4_ MNPs quenched the fluorescence of y-CDs [29]. However, the fluorescence of b-CDs with a certain concentration was quenched by y-CDs with increased concentrations, and the quenching degree was increasing (Fig. 3a). In addition, as shown in Fig. 3b, there was a huge overlay between the UV–vis absorption spectrum of y-CDs and the fluorescence spectrum of b-CDs. This means that the quenching could be caused by a fluorescence resonance energy transfer or inner filter effect between b-CDs and y-CDs [39, 40]. However, the fluorescence of b-CDs was not quenched when the probe was used to detect PTK 7. As shown in Fig. 3c, the fluorescence spectrum of b-CDs was kept at a stable value when mixed with various concentration of y-CDs-APT (20, 50, 100 ng mL^−1^). To discover the internal reason for whether the fluorescence of b-CDs was quenched or not, the fluorescence lifetime spectra of b-CDs, b-CDs + y-CDs and b-CDs + y-CDs-APT were all measured. As Fig. 3d described, when it was added to y-CDs the fluorescence lifetime of b-CDs was reduced (from 13.78 to 1.59 ns), but it showed no change after being mixed with y-CDs-APT. The fluorescence lifetime of b-CDs meanwhile, was reduced if there was an energy transfer [18, 41]. The data in Fig. 3d indicated that whether the fluorescence quenching behaviour of b-CDs by y-CDs disappeared or not was due to their separation. The separation of molecules in y-CDs and b-CDs was increased by the APT modified y-CDs and thus, destroyed their electron or energy transfer process.

All these results stated above indicate that there is no energy or photoelectron transfer process in the sensing mechanism of this probe, which means that the sensor acts in a more controlled way.

### Characterisation of Fe_3_O_4_-cDNA and y-CDs-APT

**Fig. 4:**
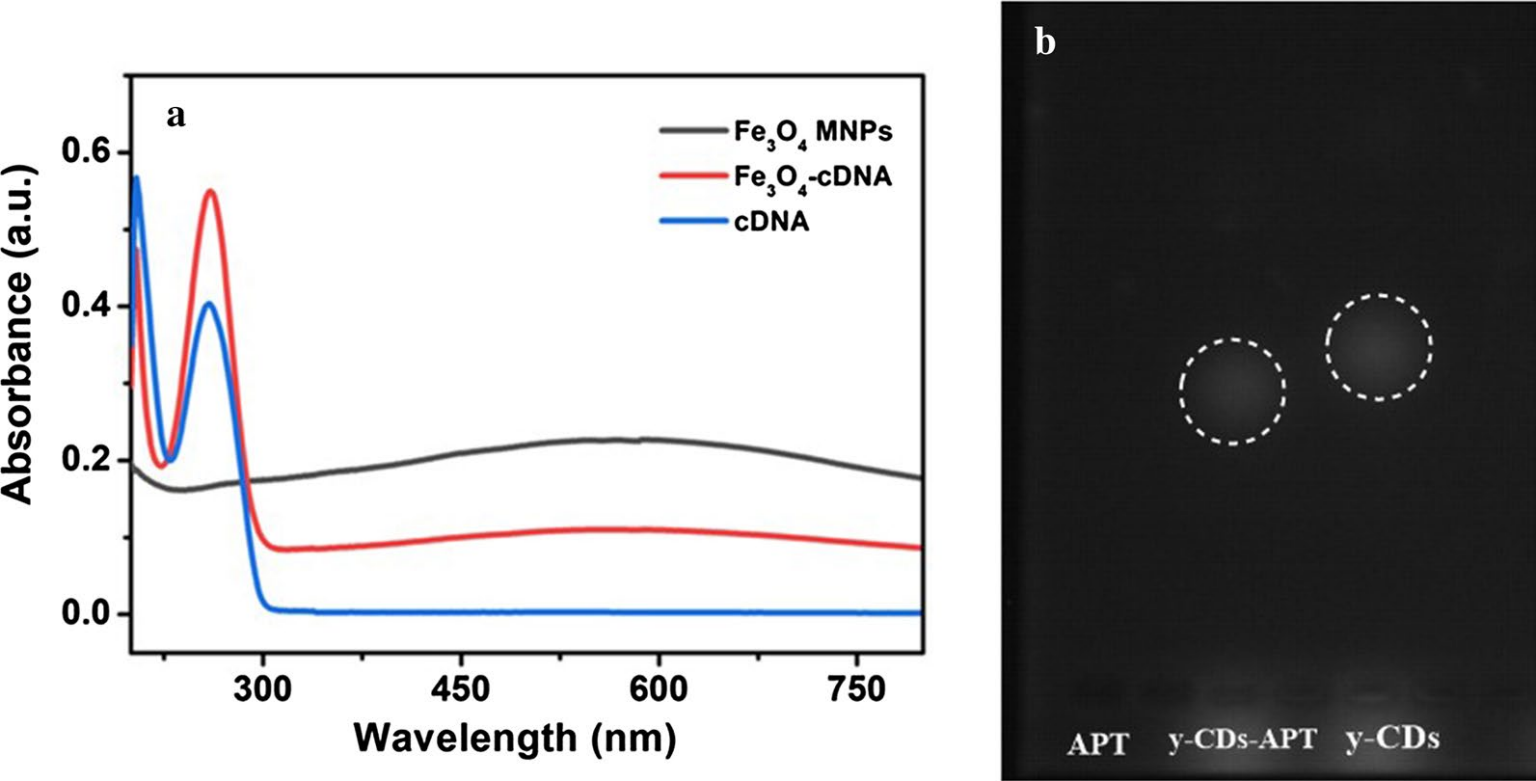
**a** The UV–vis absorption spectra of Fe_3_O_4_ MNPs, Fe_3_O_4_-cDNA and cDNA. **b** The AGE results of APT, y-CDs-APT and y-CDs

To build the probe, Fe_3_O_4_-cDNA and y-CDs-APT should be pre-composed. The conditions of this process were optimised, and the results are shown in Additional file 1: Fig. S1. As shown, when the reaction time was 60 min and the temperature was at 25 °C, the probe had a better response to PTK 7. So, 60 min and 25 °C were chosen as the optimal temperature and reaction time, respectively. Next, to prove the connection between Fe_3_O_4_ MNPs and cDNA, the UV–vis absorption spectra of Fe_3_O_4_ MNPs, Fe_3_O_4_-cDNA and cDNA were shown in Fig. 4a. As shown, Fe_3_O_4_ MNPs showed a broad absorption band at 200 ~ 800 nm, which was consistent with the previous report [31]. For Fe_3_O_4_-cDNA, a significant absorption peak was shown at ~ 260 nm as well as showing the same broad absorption band as Fe_3_O_4_ MNPs at 200 ~ 800 nm. Furthermore, the significant absorption peak at ~ 260 nm was also shown in the UV–vis absorption spectra of cDNA [19]. The results mean a successful conjunction of Fe_3_O_4_ MNPs and cDNA. To prove the conjunction of APT and y-CDs, the AGE experiment was evaluated. As Fig. 4b shows, APT displayed no fluorescence emission when under UV lamp while the fluorescence of y-CDs and y-CDs-APT (marked by white circle) were observed. The fluorescence spot of y-CDs-APT moved slower than that of y-CDs, which caused the change of shape, size and surface charge of y-CDs-APT conjugates [36]. The zeta potential of these materials was also evaluated and the results are displayed in Figure S1. The zeta potential of Fe_3_O_4_ MNPs and y-CDs both show a raised change after being attached to cDNA and APT, respectively [42]. All of these results indicate the successfully connection of Fe_3_O_4_-cDNA and y-CDs-APT.

### PTK 7 determination

**Fig. 5:**
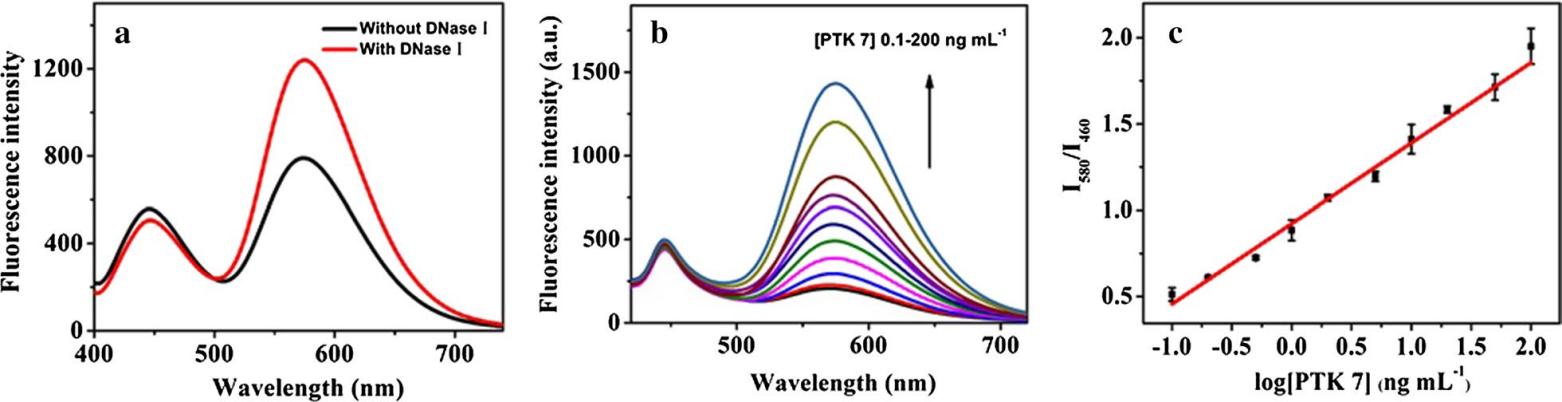
**a** Fluorescence spectra of the sensor response to PTK 7 with or without DNase I. **b** Fluorescence spectra of the probe in the presence of various concentrations of PTK 7 (in range of 0.1 − 200 ng mL^−1^). **c** The calibration curve of PTK 7 detection in range of 0.1 − 200 ng mL^−1^

To improve the sensitivity of the sensor, DNase I was added to this system, and as expected, with this addition, the developed sensor had a higher response to PTK 7. As shown in Fig. 5a, the fluorescence intensity of y-CDs (emission at 580 nm) was significantly enhanced when DNase I was added, while the fluorescence intensity of b-CDs remained nearly unchanged. The adding of DNase I enlarged the detection signals and improved the sensitivity of the probe. The results were consistent with the principle of this probe.

The reactive conditions were optimized through a single-factor test, and the results are displayed in Additional file 1: Fig. S2. The Fe_3_O_4_-cDNA and y-CDs-APT hybridisation temperature of 4 °C (Additional file 1: Fig. S2A) and 60 min reactive time were chosen as the optimum conditions. The optimum concentration (Figure S2C) and reactive time (Additional file 1: Fig. S2D) of DNase I were chosen as 20 U and 30 min, respectively. The changes in fluorescence intensity were then recorded under these optimised conditions and following the addition of various concentrations of PTK 7 (0, 0.1, 0.2, 0.5, 1, 2, 5, 10, 20, 50, 100 and 200 ng mL^−1^). As shown in Fig. 5b, with the increase of the PTK 7 concentration, the fluorescence intensity at 580 nm gradually increased while the fluorescence intensity at 460 nm remained unchanged. To find out the relationship between the PTK 7 concentrations and changing fluorescence intensity, the concentrations of PTK 7 and ratiometric fluorescence intensity (I_580_/I_460_) were analysed. As Fig. 5c shows, there was a good linear relationship between I_580_/I_460_ and the logarithm of PTK 7 concentrations (log (PTK7)) when it was in the range of 0.1–100 ng mL^−1^. The calibration equation was (n = 3): I_580_/I_460_ = 0.46653 log (PTK7) + 0.9226, r = 0.9922. The limit of detection (LOD) based on 3σ/K (where σ is the standard deviation of the blank measurement, and k is the slope of the calibration graph) was 0.016 ng mL^−1^, which was comparable or superior to most of the existing fluorescence probes for PTK 7 [43–46]. The comparison results are listed in Additional file 1: Table S1.

**Fig. 6:**
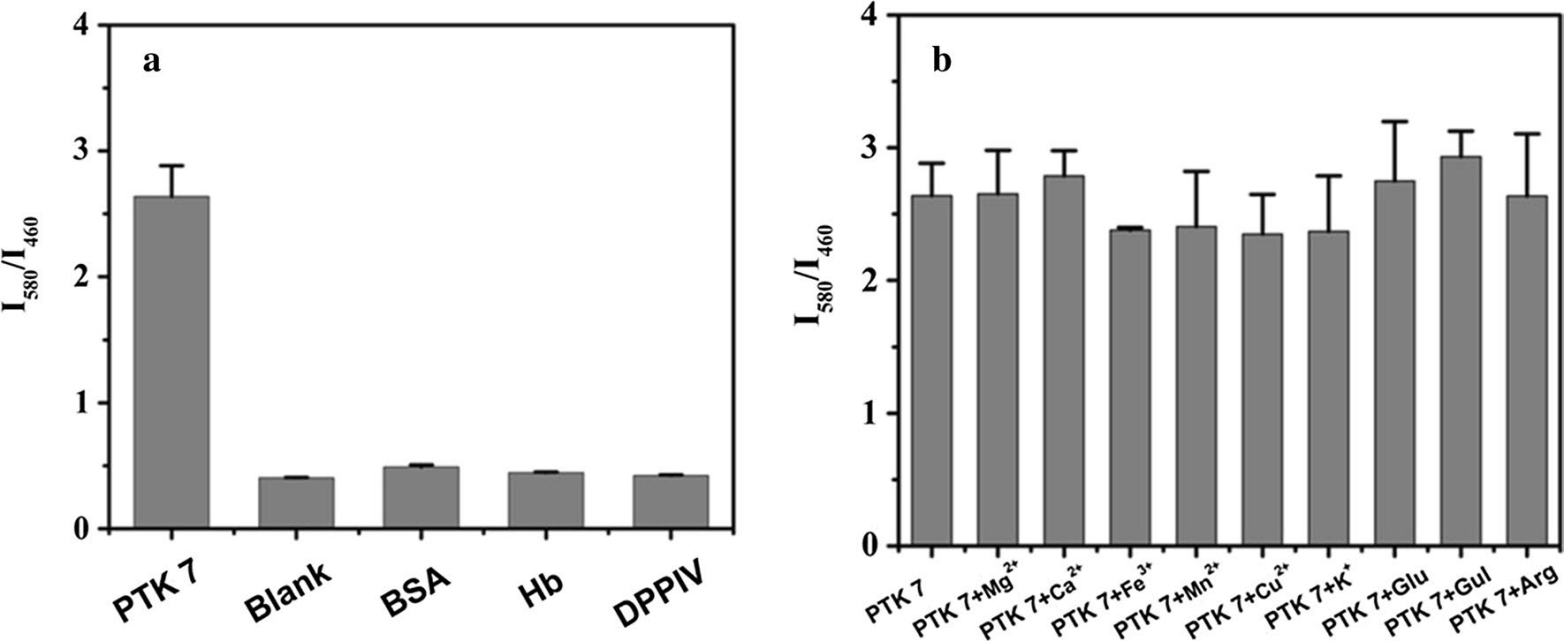
Selectivity of PTK 7 detection, and concentration of PTK 7 was 100 ng mL^−1^. **a** Concentrations of BSA, Hb and DDPIV were 1000 ng mL^−1^, while **b** the concentrations of interferents (Mg^2+^, Ca^2+^, Fe^3+^, Mn^2+^, Cu^2+^, K^+^, Glu, Gul and Arg) were also 1000 ng mL^−1^

To ensure the selectivity, accuracy and repeatability of this developed probe, the relative studies were measured. The selectivity of this probe was measured by adding interfering proteins and ions, and the results are displayed in Fig. 6. As shown in Fig. 6a, the developed probe had a high response to PTK 7 and no response to other proteins (BSA, Hb and DPPIV). In addition, the probe showed no significant difference in its PTK 7 detection after the addition, or non-addition, of high-concentration interference ions (shown in Fig. 6b). These results indicate that the developed sensor has excellent selectivity and anti-interference mechanisms. Following this, the precision and accuracy of this ratiometric fluorescence probe were measured. As shown in Additional file 1: Table S2, they were studied by assaying 0.20, 5.00 and 80.00 ng mL^−1^ of PTK 7 in three separate runs. The intra-day and inter-day relative standard deviations (RSDs) were in the range of 3.6–10.2% and 1.8–10.9%, respectively, while the accuracies were all in the range of 97.5–101.5%. The results suggest that the ratiometric fluorescence probe has promising potential for the detection of PTK 7, and could be used for determination in real samples.

### Detection in real samples

**Fig. 7:**
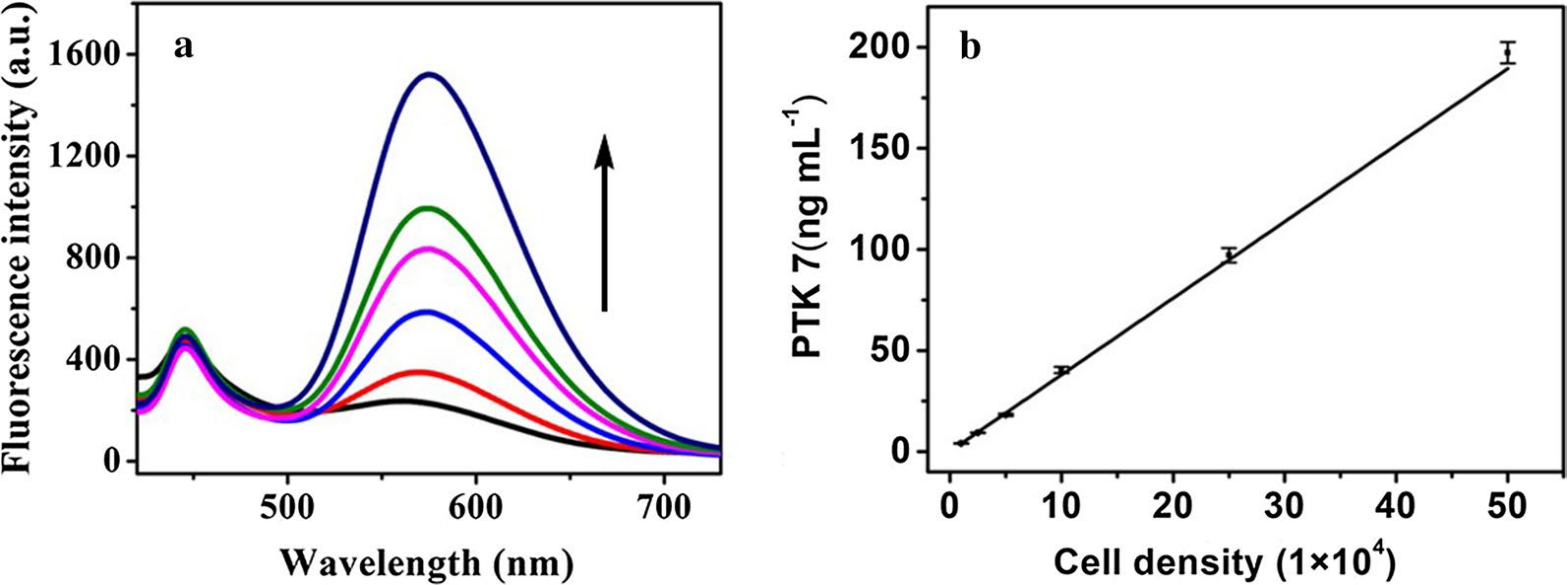
**a** Fluorescence spectra of the probe in the presence of various cell densities of MCF-7 cells (in range of 1 × 10^4^ to 5 × 10^5^ per mL). **b** The calibration curve of PTK 7 detection in MCF-7 cells in range of 1 × 10^4^–5 × 10^5^ per mL

According to the functions of this developed probe, MCF-7 cells and human serum were used as the practical examples. Taking MCF-7 cell determination as an example, it could be seen that the intensity of the fluorescence of this developed probe increased with the rising cell density (Fig. 7a). Furthermore, it was observed in Fig. 7b that there was a good linear relationship between PTK 7 concentrations and MCF-7 cell density in the range of 1 × 10^4^ to 5 × 10^5^ per mL (C _PTK 7_ = 3.784 C _cell density_ + 0.2126, r = 0.9989, n = 3, and the unit of cell density was 1 × 10^4^ per mL). Next the recovery of PTK 7 during MCF-7 cell determination were evaluated, and the results are displayed in Additional file 1: Table S3. As shown, the recoveries for the detection of PTK 7 in MCF-7 cells were between 94.2% and 105.3%. The RSDs were from 2.7 to 4.9%. Additionally, the developed sensor was used to detect PTK 7 in human serum, with the recovery results shown in Additional file 1: Table S4. Additional file 1: Table S4 shows that the PTK 7 recoveries in human serum were between 90.2 and 96.4% while the RSDs were from 3.9 to 6.7%.

All of the results prove that this multi-nanomaterial based ratiometric fluorescence sensor has excellent sensitivity and selectivity, and that the probe could be used to detect PTK 7 in MCF-7 cells and human serum. These results indicate that the developed probe has great potential for use in clinical applications.

## Conclusion

In summary, we successfully developed a ratiometric fluorescence probe based on multi-nanomaterials (b-CDs, y-CDs and Fe_3_O_4_ MNPs) and an aptamer for PTK 7 detection in this work. In the absence of PTK 7, a y-CDs modified aptamer and cDNA modified Fe_3_O_4_ MNPs were assembled together, the fluorescence of y-CDs was quenched by Fe_3_O_4_ MNPs but recovered by adding PTK 7, and the ratiometric fluorescence probe was built by using b-CDs as the internal reference. Additionally, DNase Ι was added in order to set up a loop amplifier to enhance the sensitivity of this probe. This developed sensor exhibits a LOD of up to 0.016 ng mL^−1^, which outperforms previous methods and is highly sensitive and selective in its determination of PTK 7. Furthermore, this sensitive and selective probe was successfully used to detect PTK 7 in both MCF-7 cells and human serum. This thus shows a huge potential for its application in clinical practice.

## Supplementary Information

**Additional file 1: Figure S1.** The zeta potential of Fe_3_O_4_ MNPs, Fe_3_O_4_-cDNA, y-CDs and y-CDs-APT. **Figure S2.** The optimum reactive conditions of PTK 7 determination. The temperature (**A**) and the reactive time (**B**) of Fe_3_O_4_-cDNA and y-CDs-APT hybridization reaction, the concentration (**C**) and the reactive time (**D**) of DNase I. All measurements were performed by single-factor test. **Table S1.** Comparison of the probe performance of this Work with those previously reported sensing methods. **Table S2.** Precision and accuracy of PTK 7 determination. **Table S3.** Recoveries for PTK 7 in MCF-7 cells (5 × 10^4^) determination. **Table S4.** Recoveries for PTK 7 in human serum determination.

## Data Availability

All data analyzed during this study are included in this published article.

## Abbreviations

PTK 7: Protein tyrosine kinase 7
CDs: Carbon dots
LOD: Limit of detection
MNPs: Magnetic nanoparticles
EDC: Diethylenetriamine, *N*-(3-dimethylaminopropyl)-Nethylcarbodiimide hydrochloride
NHS: *N*-hydroxysuccinimide
GABA: 4-Aminobutyric acid
OPD: *o*-Phenylenediamine
TEM: Transmission electron microscopic images
RSDs: Relative standard deviations
