# Supplementary material for: Multi-carbon dots and aptamer based signal amplification ratiometric fluorescence probe for protein tyrosine kinase 7 detection
Source: J Nanobiotechnology. 2021 Feb 15;19:47. doi: 10.1186/s12951-021-00787-7 (PMC7885398; doi:10.1186/s12951-021-00787-7)
Supplement: Supplementary file 1 — Additional file 1: Figure S1. The zeta potential of Fe3O4 MNPs, Fe3O4-cDNA, y-CDs and y-CDs-APT. Figure S2. The optimum reactive conditions of PTK 7 determination. The temperature (A) and the reactive time (B) of Fe3O4-cDNA and y-CDs-APT hybridization reaction, the concentration (C) and the reactive time (D) of DNase I. All measurements were performed by single-factor test. Table S1. Comparison of the probe performance of this Work with those previously reported sensing methods. Table S2. Precision and accuracy of PTK 7 determination. Table S3. Recoveries for PTK 7 in MCF-7 cells (5 × 10^4^) determination. Table S4. Recoveries for PTK 7 in human serum determination. [file 12951_2021_787_MOESM1_ESM.docx]

**Additional file**

Multi-carbon Dots and Aptamer Based Signal Amplification Ratiometric Fluorescence Probe for Protein Tyrosine Kinase 7 Detection

Yunsu Ma, Yuan Wang, Yongjie Liu, Lujia Shi, Dongzhi Yang*

School of Pharmacy, Xuzhou Medical University, Xuzhou, Jiangsu 22004, PR China

Email address of the corresponding author：dongzhiy@xzhmu.edu.cn


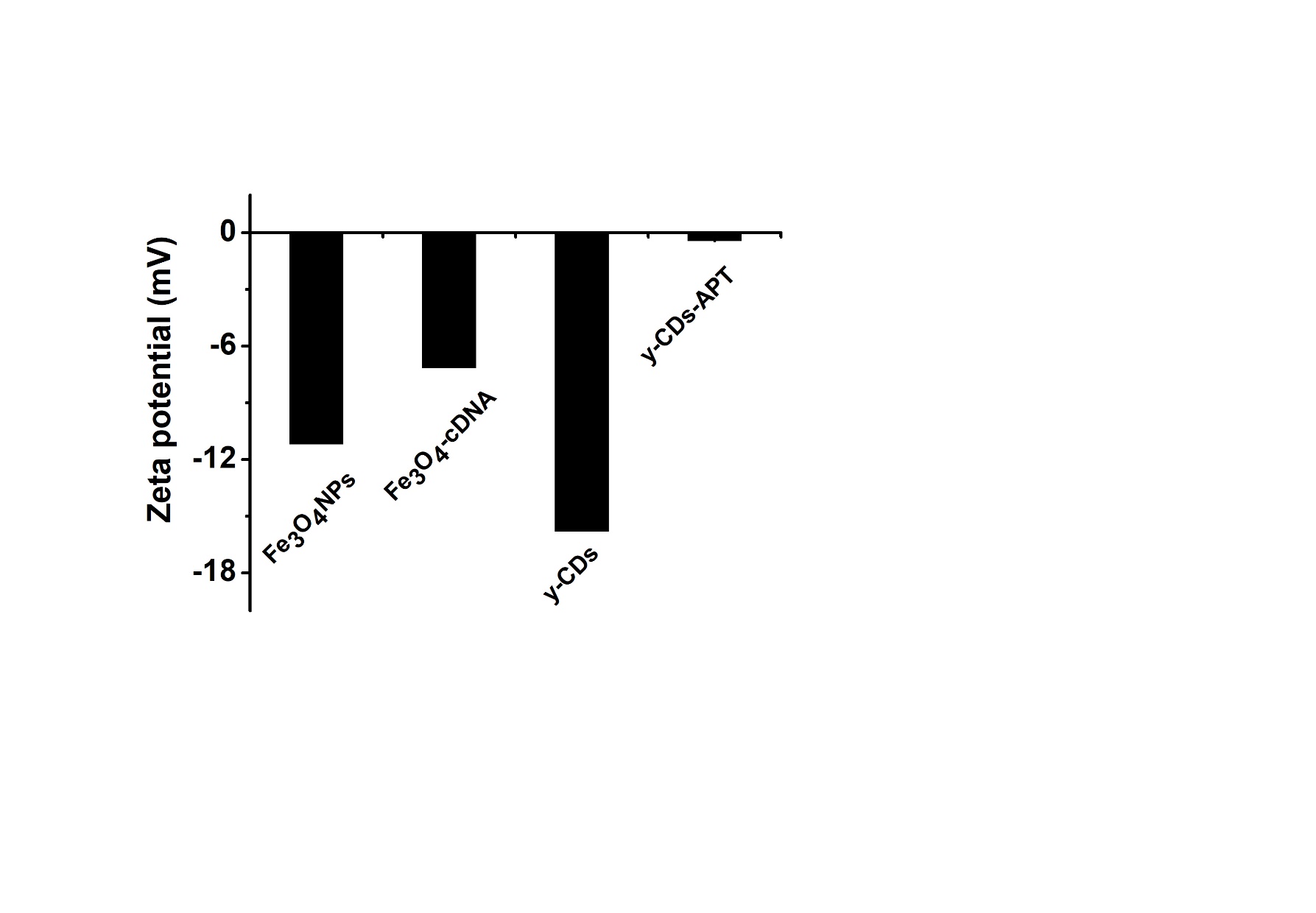


**Figure S1 The zeta potential of Fe_3_O_4_ MNPs, Fe_3_O_4_-cDNA, y-CDs and y-CDs-APT.**


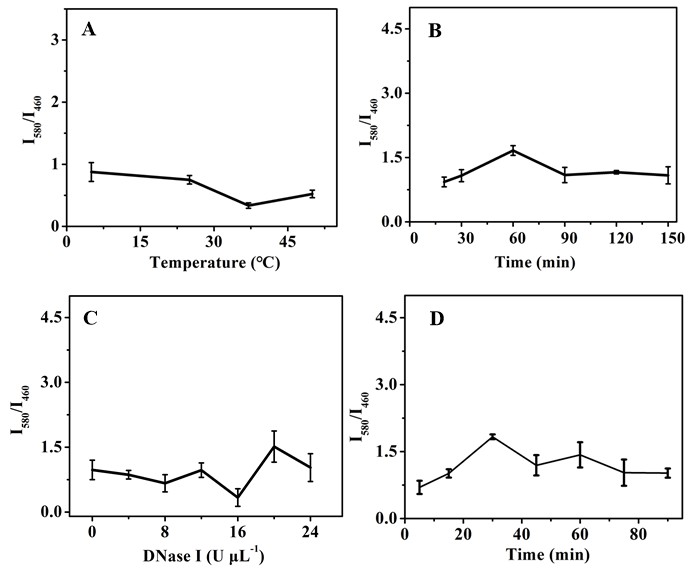


**Figure S2 The optimum reactive conditions of PTK 7 determination. The temperature (A) and the reactive time (B) of Fe_3_O_4_-cDNA and y-CDs-APT hybridization reaction, the concentration (C) and the reactive time (D) of DNase Ⅰ. All measurements were performed by** **single-factor test.**

**Table S1.** Comparison of the probe performance of this Work with those previously reported sensing methods

| ref. | Detection method | LOD |
| --- | --- | --- |
| [34](#_ENREF_1) | Fluorescence method | 12 pM (0.3132 ng mL^-1^) |
| [35](#_ENREF_2) | Electrochemical method | 372 fM (0.009 ng mL^-1)^ |
| [36](#_ENREF_3) | Fluorescence method | 102.8 pM (2.68 ng mL^-1^) |
| [37](#_ENREF_4) | Fluorescence method | 1 pM (0.0261 ng mL^-1^) |
| This work | Ratiometric fluorescence method | 0.016 ng mL^-1^ |

**Table S2.** Precision and accuracy of PTK 7 determination

| Analyte name | Nominal concentration  (ng mL^-1^) | Intra-run (n=3) | | | Inter-run (n=3) | | |
| --- | --- | --- | --- | --- | --- | --- | --- |
|  |  | Found  (ng mL^-1^) | RSD | Accuracy | Found  (ng mL^-1^) | RSD | Accuracy |
| PTK 7 | 0.20 | 0.198 | 10.2% | 99.0% | 0.196 | 10.9% | 98.0% |
|  | 5.00 | 5.07 | 7.6% | 101.5% | 4.88 | 8.4% | 97.5% |
|  | 80.00 | 79.58 | 3.6% | 99.5% | 78.96 | 1.8% | 98.7% |

**Table S3** Recoveries for PTK 7 in MCF-7 cells (5×10^4^) determination

| Sample | Spiked  (ng mL^-1^) | Found  (ng mL^-1^) | Recovery | RSD |
| --- | --- | --- | --- | --- |
| MCF-7 Cells | 0.00 | 19.11 | -- | -- |
|  | 20.00 | 39.08 | 99.4% | 2.9% |
|  | 40.00 | 42.12 | 105.3% | 2.7% |
|  | 60.00 | 56.52 | 94.2% | 4.9% |

**Table S4** Recoveries for PTK 7 in human serum determination

| Sample | Spiked  (ng mL^-1^) | Found  (ng mL^-1^) | Recovery | RSD |
| --- | --- | --- | --- | --- |
| serum | 0 | ND | - | - |
|  | 0.10 | 0.0962 | 96.3% | 4.6% |
|  | 0.20 | 0.180 | 90.2% | 3.9% |
|  | 0.30 | 0.289 | 96.4% | 6.7% |
